# Parent Involvement in the Getting Ready for School Intervention Is Associated With Changes in School Readiness Skills

**DOI:** 10.3389/fpsyg.2018.00759

**Published:** 2018-05-31

**Authors:** Maria Marti, Emily C. Merz, Kelsey R. Repka, Cassie Landers, Kimberly G. Noble, Helena Duch

**Affiliations:** ^1^Heilbrunn Department of Population & Family Health, Mailman School of Public Health, Columbia University, New York City, NY, United States; ^2^Neurocognition, Early Experience and Development Lab, Neuroscience & Education, Teachers College, Columbia University, New York City, NY, United States

**Keywords:** school readiness, Head Start, parent involvement, early intervention, prevention

## Abstract

The role of parent involvement in school readiness interventions is not well-understood. The Getting Ready for School (GRS) intervention is a novel program that has both home and school components and aims to improve early literacy, math, and self-regulatory skills in preschool children from socioeconomically disadvantaged families. In this study, we first examined associations between family characteristics and different indices of parent involvement in the GRS intervention. We then examined associations between parent involvement and change in children's school readiness skills over time. Participants were 133 preschool children attending Head Start and their parents who participated in the GRS intervention during the academic year 2014–2015. Parent involvement was operationalized as attendance to GRS events at the school, time spent at home doing GRS activities, and usage of digital program materials, which included a set of videos to support the implementation of parent-child activities at home. Although few family characteristics were significantly associated with parent involvement indices, there was a tendency for some markers of higher socioeconomic status to be linked with greater parent involvement. In addition, greater parent involvement in the GRS intervention was significantly associated with greater gains in children's early literacy, math, and self-regulatory skills. These findings suggest that parent involvement in comprehensive early interventions could be beneficial in terms of improving school readiness for preschoolers from disadvantaged families.

## Introduction

Children from socioeconomically disadvantaged families tend to enter school behind their peers in terms of pre-academic and self-regulatory skills (Bradley and Corwyn, 2002; Noble et al., 2005). This lack of early preparation for school leads to an academic achievement gap that widens over time (West et al., 2001; Reardon, 2011), putting children from disadvantaged families at risk for school failure, dropout, and markedly fewer professional opportunities in adulthood. Family-oriented early interventions that support parents' capacity to promote child development can help reduce this gap in school readiness. Active engagement of parents is essential to the successful implementation of such interventions (Shaw et al., 2006). However, parental participation remains a challenge for many programs (Patterson and Chamberlain, 1994). In addition, it is unclear how parents engage with different intervention components (e.g., in-person workshops, use of program materials at home) and whether or not these components are differentially associated with children's school readiness outcomes.

In this study, we explore these issues through the lens of Getting Ready for School (GRS), a novel early intervention targeting teachers and parents that supports the development of school readiness skills in preschool children from socioeconomically disadvantaged backgrounds (Noble et al., 2012; Marti et al., 2018). GRS is unique in its focus on all three of the main school readiness domains (early literacy, math, and self-regulation) and its equal emphasis on teachers and parents as agents of change. The home component, which is the focus of the current study, promotes parental use of activities that support the development of literacy, math, and self-regulation skills. Parents are provided with activities to integrate into day-to-day, play-time interactions with their children. To support the implementation of these activities, GRS offers a series of workshops, parent-child activities in the classroom, videos demonstrating how to implement the activities, and a bi-weekly parent-teacher letter.

By better understanding how parents engage in early interventions such as GRS and the implications of parent involvement for improving children's school readiness, researchers can begin to improve delivery models and increase the effectiveness of family-oriented early interventions. In the present study, we focused on parent involvement with different components of GRS. Parent involvement with GRS was operationalized as (1) attendance to workshops and classroom events, (2) time spent using GRS activities at home, and (3) usage of the digital program materials. We explored how family characteristics relate to parent involvement with GRS, and the associations between parent involvement and children's growth in school readiness skills across the preschool year.

### Background

Parental involvement in children's early development and education includes initiating learning activities in the home, building positive relationships, engaging in preschool activities, and communicating with teachers (Epstein, 2001). Ecological theories of parent involvement acknowledge its multidimensional nature and recognize that parents are involved across different contexts, such as home and school (Seginer, 2006; Garbacz et al., 2015; McWayne et al., 2015). Home-based parent involvement refers to the ways in which parents support learning by playing and interacting with their children, providing learning activities, and offering materials that support development. School-based parent involvement includes participation in school-based events like field trips, activities, and workshops, or volunteering in the classroom.

Parent involvement has the potential to bolster school readiness in children from disadvantaged families and reduce the income-achievement gap. In fact, home- and school-based parent involvement facilitate pre-academic skills and social competence during preschool (Miedel and Reynolds, 1999; Fan and Chen, 2001; Dearing et al., 2004; McWayne et al., 2004; Gonzalez-DeHass et al., 2005; Van Voorhis et al., 2013). A longitudinal study that followed children from kindergarten to 5th grade showed that increased school-based parent involvement (e.g., attending parent–teacher conferences, participating in school activities, or volunteering in the classroom) predicted improved child literacy skills (Dearing et al., 2006). Similar results have been reported for home-based parent involvement. In Head Start families, parent involvement at home (e.g., self-reports of reading to the child or providing learning activities) was associated with positive growth in children's attention, persistence, motivation to learn, and receptive vocabulary; and decreased problem behaviors (Fantuzzo et al., 2004).

In the last decade, many early interventions have been developed to support parenting practices and parent involvement in early education. A range of different interventions have demonstrated positive impacts on school readiness in preschool children, including those that promote positive parenting practices and parent-child relationships; those that promote home learning activities and effective teaching strategies; and those that strengthen parent-teacher partnerships (Bierman et al., 2017). These programs have used various methods of connecting with parents, including face-to-face intensive individual coaching (Sheridan et al., 2011; Landry et al., 2012) and regular parenting groups (Mendez, 2010; Brotman et al., 2011). Often, program content is also delivered through videotaped demonstrations of parent-child interactions (Whitehurst et al., 1994; Webster-Stratton, 1998; Landry et al., 2012), written information, and/or enrichment materials and activities for home use with children (Bierman et al., 2008a,b).

Embedded in this literature is a call to better understand and improve parent involvement in early interventions (Bierman et al., 2017). Indeed, a common challenge in family-oriented interventions is getting parents involved and sustaining parent involvement throughout the course of the intervention (Fishel and Ramirez, 2005; Shaw et al., 2006; Gross et al., 2009; Halgunseth et al., 2009). These challenges are especially relevant for programs that rely on group-based training or workshops. Recent studies on preventive interventions for behavior problems reported that only about 30–48% of targeted families participate in at least one workshop (Heinrichs et al., 2005; Garvey et al., 2006). Slightly more encouraging, results from three school readiness interventions indicated that about 60–80% of families participate in parent sessions. In terms of sustaining parent involvement, parents have been found to attend only between 20 and 35% of offered parent workshops (Miedel and Reynolds, 1999; Mendez, 2010; Dawson-McClure et al., 2015).

Few studies of parent involvement have focused on parent use of written and/or internet-based program materials, such as those focused on activities to support child learning and development at home (e.g., videos that depict parent-child learning activities). Interventions that use home visiting models suggest high levels of parent involvement with program materials. For example, almost 90% of parents' participating in REDI-P engaged with program materials at home on a weekly basis as observed by home visitors (Bierman et al., 2015). However, use of program materials in workshop-based interventions tends to be lower. For example, although most parents participating in *The Companion Curriculum* used program materials (i.e., parent-child activities) at some point across the intervention, only 50% of parents used them once a week (Mendez, 2010). Thus, research is needed on how much parents use intervention-related program materials meant to enhance home-based parent involvement and which family characteristics might facilitate parental use of program materials.

Various family characteristics might be expected to influence parent involvement both at home and at school (Grolnick et al., 1997; Cooper et al., 2010). Studies examining family sociodemographic characteristics associated with attendance to workshops have been inconsistent. For example, unemployed parents had greater attendance at *ParentCorps* workshops while the least educated families attended fewer sessions (Dawson-McClure et al., 2015). In a study of Head Start parents, there were no differences in parent participation in *The Companion Curriculum* workshops by parental education level (Mendez, 2010). Single parenthood has also been associated with lower attendance in some prevention programs (Reyno and McGrath, 2006; Baker et al., 2011) yet others have not found these associations (Orrell-Valente et al., 1999). Similar mixed findings have emerged for minority status. Some research suggests that African-American and Hispanic/Latino families have lower levels of attendance, even when controlling for SES factors (Nix et al., 2009; Baker et al., 2011). Nonetheless, research with a nationally representative sample of Head Start parents found that Hispanic/Latino and Asian families showed greater school-based parent involvement than other ethnic groups (Hindman et al., 2012). Thus, attendance for minority families may depend on factors such language needs or cultural appropriateness of interventions (Murry et al., 2004).

To our knowledge, there is no research that has examined the associations between family characteristics and indices of parent involvement in early interventions other than attendance. Some of the factors associated with attendance may also play a crucial role in how parents spend time at home using program materials. For example, being an immigrant or single parent has been associated with lower levels of home-based parent involvement in Head Start (e.g., reading books, playing games, teaching about letters, words, and numbers) (Hindman et al., 2012). Research has also documented socioeconomic differences in the amount of developmentally relevant time (e.g., playing and reading) parents spend with children (Guryan et al., 2008; Kalil et al., 2012). Yet, family characteristics may be differentially associated with school-based parent involvement (e.g., attendance and participation) and home-based parent involvement (e.g., use of program activities at home) in family-oriented school readiness interventions such as GRS. For example, despite Latino Head Start mothers being highly involved in school activities, they reported less home-based involvement compared to other ethnic groups (Hindman et al., 2012). Therefore, research is needed to address this gap in the literature.

Another important research question is the extent to which parent involvement may explain variability in children's school readiness outcomes following early interventions (Reynolds et al., 1996, 2017). Parent involvement has been found to moderate the impact of behavioral interventions (Breitenstein et al., 2012; Gross et al., 2014; Patel et al., 2016). For example, parent attendance and completion of between-session activities have been associated with treatment gains in parenting skills and mental health (Nix et al., 2009; Clarke et al., 2015). In addition, more frequent parent attendance at *ParentCorps* workshops was associated with greater knowledge of positive parenting practices and effective behavior management strategies (Brotman et al., 2011, 2013, 2016). Research on school readiness interventions has yielded similar findings. For instance, more frequent attendance to *The Companion Curriculum* workshops was associated with more optimal parent-teacher relationships (Mendez, 2010). However, previous work has not detected effects of parent attendance on children's school readiness outcomes or explored the effects of parents' use of program materials at home on children's school readiness outcomes. Certain interventions, like *Head Start REDI-P*, which used a home visiting model, have demonstrated that the frequency and quality of how parents use home learning materials was associated with academic performance in kindergarten (Bierman et al., 2015). Thus, it is important to examine to what extent parental attendance to workshops and time spent at home using intervention-related activities is associated with children's school readiness outcomes following early interventions. This type of formative research is critical to improving strategies that support parent involvement and thus potentially increasing the impact of interventions.

### Present study

In the present study, we examined the associations between family characteristics and parent involvement in GRS and the associations between parent involvement and children's gains in school readiness skills across the preschool year. Parent engagement in GRS was operationalized as (1) attendance to workshops and classroom events, (2) time spent using GRS activities at home, and (3) usage of digital content. Thus, this study offers a comprehensive picture of different ways in which parents may engage in school readiness interventions.

We had three specific research questions:

1. What are the rates of parental attendance, time spent doing activities at home, and usage of digital content? Given that relatively little is known about patterns of parent involvement in early interventions, it was crucial to first explore and describe how parents engage with the different components of GRS.
2. Which family characteristics (e.g., income-to-needs ratio, parental education, parental employment, language, father's presence, country of origin, and use of social services) are associated with parent involvement in GRS? Because findings from previous research have been inconsistent, we did not have any formal hypotheses about these associations.
3. Which indices of parent involvement predict children's school readiness outcomes following the intervention? We hypothesized that children whose parents attended more events and/or spent more time using GRS activities would show more growth in self-regulatory and pre-academic skills across the preschool year.

## Methods

### Participants

Children and their parents (*N* = 147) at four Head Start centers in New York City were invited to participate in the GRS intervention. Overall, 90.5% (*n* = 133) of families consented to participate in the evaluation; 8.8% (*n* = 13) did not return the consent form; and one family refused to participate. Informed consent and all study procedures were approved by the Columbia University Medical Center Institutional Review Board. Of the respondents who participated in the study, 88.24% were mothers, 5.88% were fathers, and 2.94% were grandmothers. Most caregivers spoke English (51.9%) or Spanish (38.3%), and 9.8% spoke Chinese. Child age at baseline ranged from 40 to 57 months (*M* = 50.28; *SD* = 4.12), and child sex was evenly distributed (51% male). The majority of children were Hispanic/Latino (72%). Parent education ranged from less than high school (25.49%) to graduate degree (13.73%); they had an average of 12.11 years of education (*SD* = 3.09). Average family income-to-needs ratio was below the poverty line (*M* = 0.85, *SD* = 0.79). In more than two thirds of families, the father was present in the child's life (73.53%). Full child and family demographics (collected via school records and parent questionnaires) are provided in Table 1.

**Table 1:** Descriptive statistics for sample characteristics.

	***M*(*SD*) or %**
Child sex: male	51.40
Child age in months	50.28 (4.12)
Child ethnicity: Hispanic/Latino	72.00
Child race	
White	8.84
Black or African American	8.84
Asian	5.44
American Indian	1.36
Other	22.45
Biracial	0.68
Not reported	21.77
Respondent	
Mother	88.24
Father	5.88
Grandmother	2.94
Not reported	2.94
Respondent education (years)	12.11 (3.09)
Less than high school diploma	25.49
High school diploma or GED	35.29
Some college	13.73
College degree	13.73
Not reported	11.76
Family income-to-needs ratio	0.85 (0.79)
Father present: yes	73.53
Marital status (^*^)	
Two-parent household	55.07
One-parent household	40.58
Other	2.90
Not reported	1.45
Respondent US born (^*^)	36.23
Respondent employment (^*^)	
Full-time	28.99
Part-time	14.49
Homemaker	28.99
Not working	26.09
Not reported	1.45
Social services (^*^)	
SNAP	47.83
WIC	31.88
TANF	7.25

Child characteristics were collected from school records. At baseline, families reported on maternal and paternal education and income. Percentages are based on 102 families that completed these questionnaires. At posttest, families were administered a survey that included extra demographic questions marked with

* *. For these variables, percentages are based on 69 families that returned the survey*.

*U.S., United States; SNAP, Supplemental Nutrition Assistance Program; WIC, Women, Infants and Children; TANF, Temporary Assistance for Needy Families*.

### Intervention procedures

The GRS intervention was implemented in 4 Head Start centers from October 2014 to June 2015. All centers offered a full day program and followed the Teaching Strategies Creative curriculum (Dodge et al., 2002). A total of seven classrooms received the GRS intervention. Selection of centers and classrooms receiving GRS was not randomized. School directors chose which classrooms participated in the intervention. This was the first time schools, teachers, and families had participated in GRS.

GRS was originally designed as an intervention for parents in low and middle income countries to promote math and literacy skills through activities and bi-weekly parent workshops. A small randomized study with Head Start families demonstrated improvements in math skills over and above a Head Start-as-usual experience (Noble et al., 2012). GRS was subsequently enhanced by developing a classroom curriculum, adding activities to support children's self-regulation, and enhancing the parent component. A 2-year collaborative development process of the activities that included parents and teachers from partner Head Start centers ensured that new materials were culturally appropriate.

The resulting enhanced model is a preschool intervention targeting children's development of language, literacy, mathematics, and self-regulation skills by enhancing the home and classroom environments. The GRS intervention offers teachers and parents a set of activities that are meant to be integrated into playful time. The activities are designed to promote joint engagement and encourage parents and teachers to ask open-ended questions and follow the children's lead. The classroom component consists of a series of supplemental classroom activities designed to promote literacy, math, and self-regulation. These classroom activities are organized into nine units following a developmentally-appropriate sequence, and include tools to help teachers choose activities that will easily integrate into chosen themes or target skills (see Table 2). Teachers are asked to implement two to three GRS activities each day, for a total of about 30–45 min. Throughout program implementation, teachers receive a full-day introductory training and individualized support from a classroom coach, who was also a member of the research team. Teachers also receive biweekly coaching from a GRS staff member who guides them in implementing the GRS activities in the classroom following the principles of Practice Based Coaching (PBC; Head Start, 2015). During coaching meetings, teachers planned the implementation of GRS activities and reflected on previous activities implemented. On alternate weeks, coaches observed classroom activity implementation, with modeling and live coaching support accompanying these sessions. All classrooms coaches either held or were pursuing a Masters or PhD degree in educational or psychological fields and received rigorous training and supervision by the project principal investigators (Marti et al., 2018).

**Table 2:** Scope and sequence of GRS parent component.

**Unit**	**Math**	**Literacy**	**Self-regulation**
1	Counting to determine quantity, counting sets of objects up to 5, comparing groups with “more” and “less”	Letter recognition, letter-sound correspondence, print conventions	Paying attention, self-control skills
2	Sorting objects into groups, counting sets of objects up to 5, recognizing numbers 1–5	Print conventions, sound and letter recognition name, identifying rhymes, letter-sound correspondence	Recognizing, managing, and talking about feelings
3	Recognizing numbers 6–10, counting sets of objects up to 10, sorting objects, comparing groups of objects	Expressive language, creating stories, phonological awareness, identifying rhymes	Talking about feelings of characters in stories
4	Recognizing numbers 6–10, counting sets of objects up to 10	Identifying favorite words, expressive language, identifying rhymes, creating stories	Working memory, identifying and talking about feelings
5	Measuring length and weight, comparing objects, making predictions	Letter recognition, print conventions, expressive language, writing, letter-sound correspondence	Paying attention, working memory, self-control, managing emotions
6	Identifying, recognizing, and counting shapes; sorting objects by shape, color, and size	Expressive language, rhyming, story sequencing	Thinking about feelings, making lists to help children follow directions, remember, and complete tasks
7	Identifying and making patterns	Identifying words, expressive language, creating stories, story sequencing	Working memory, paying attention using the “if-then” rule
8	Practicing simple addition	Reading and writing, expressive language, recognizing words	Taking turns, cooperation and working together
9	Practicing simple subtraction, Learning about zero	Reading and writing, story sequencing, expressive language	Paying attention, discussing feelings about Kindergarten

*This sequence of units corresponds to that which was followed by teachers in the GRS intervention*.

The GRS enhanced home component uses three delivery models to support parent involvement: print, in-person and digital. At the beginning of the academic year, parents receive a color-printed workbook and a set of materials to complete GRS activities from the book. Parents are offered learning activities, organized into nine units following a developmentally appropriate sequence (see Table 2). Activities can be easily completed with everyday household materials, are meant to be integrated into everyday family life, and include activities that can be done in the home or the neighborhood. For example, children can practice counting, sorting, and adding by playing “what belongs? what does not?” where children take different objects they have at home and they have to sort them following different rules. Another example is “hunt for letters” where children practice letter and sound recognition by going for a walk and finding the letters of their names. Children and parents can also practice self-regulation skills playing “let's play freeze” as they dance their favorite song or talk about different feelings as they engage in “make a feelings bookmark” using pictures from newspapers and magazines. The book also offers twelve extra tips on self-regulation such as how to help children stay calm, how to build the child's helping skills, how to use think-aloud strategies, or how to talk about feelings. Self-regulation activities and tips were developed in partnership with the team of investigators who designed the Social Emotional Cognitive Understanding and Regulation (SECURe) curriculum (Jones et al., 2014a,b). Activities in the parent book are aligned with GRS classroom activities. Materials are available in English, Spanish, and Mandarin, and were designed for a 3rd grade reading level. In addition, in collaboration with a program coach, teachers give parents a biweekly letter that assigns activities from the program activity book to be done at home, which are parallel to the classroom activity schedule.

GRS provides one tablet computer per classroom, which includes a collection of videos depicting real parents demonstrating how to implement program activities at home. Tablets are available in every classroom and caregivers can check them out for a week, much like a library book. Videos are available in Spanish and English and are designed with special attention to culturally diverse and low-literacy parents. In addition, a program website with all videos and print lessons is available to all parents.

In addition to the print and online materials, 11 GRS events are offered for parents to attend (one orientation, eight workshops through the year, and two classroom parties) which aim to support parents' implementation of the activities at home (see Table 3). At the beginning of the year, parents are invited to an orientation where they receive the print materials. If parents are unable to attend, materials are left in children's cubbies with a pamphlet that contains information about the program. During this first meeting, team members define GRS and facilitate hands-on group activities so that parents can experience the GRS materials. The team then offers a series of eight parent workshops throughout the school year: three in the fall, four in the winter, and one in the spring. Each workshop lasts 1 h and includes didactics, modeling, role-plays, discussions, and group activities designed to teach math, literacy, and self-regulation skills. Workshops are led by team members who hold at least a Bachelors or Master's degree in an education or mental health field. Group leaders are trained by the GRS program director and a postdoctoral fellow and followed a facilitator's guide. To assess program fidelity and program efficacy (Matthews and Hudson, 2001), group leaders filled out a form after each session comprised by 11 items rating adherence to the program, group leader self-efficacy, and overall participant engagement on a scale from 1 (*strongly disagree*) to 5 (*strongly agree*). On average, self-reported fidelity to the intervention ranged from 3.72 to 4.90 (*SD* = 0.44).

**Table 3:** GRS events offered at the school.

**Month**	**Theme**	**Content**	**Average parent attendance %**
October	Orientation	Program presentation and materials exploration	38
December (Weekly)	Self-regulation workshop: What is and how can help at home and school.	Overview of different components of self-regulation. Focus on emotion recognition and expression	31
	Literacy workshop: Getting ready to read and write.	Overview of pre-literacy skills that support development of reading and writing. Letter recognition and phonological awareness	17
	Math workshop: numbers and counting	Overview of math skills and expectations for preschoolers. Counting, one-to-one correspondence, number recognition, simple addition.	17
February	GRS party	Parent and children play 3 activities that focus on letter identification, counting, emotion recognition	31
March-April (Weekly)	Literacy workshop: Asking questions and making stories.	Build vocabulary and creativity using open-ended questions and making stories together.	16
	Math workshop: Measurement, shapes and patterns.	Measurement in preschool: using non-standard units of measurement. Patterns: learning to make predictions	19
	Self-regulation workshop: Understanding emotions.	How to support children to identify their emotions and talk about them. Pretend play, story making.	17
	Self-regulation workshop: Managing emotions and behavior.	Emotion regulation. How to prevent and manage conflicts.	12
May	GRS party	Parent and children play 3 activities that focus on rhyme identification, making shape patterns, working memory	30
June	Transition: prepare your children for kindergarten.	How to support the transition to kindergarten before, the first day, and the first weeks.	16

At different points in the year, two 1-h classroom parties take place at dismissal time. GRS facilitators prepare three GRS activities (one from each domain) and invite parents to play with their children while facilitators and teachers supported parent involvement with children by reflecting on positive interaction and using role model when needed. At the end of each party, parents, children, teachers, and the GRS team do an activity together, led by one of the GRS facilitators (e.g., read a poem and find rhyming words). Classroom parties conclude with raffles to win art supplies and child magazines. Table 3 presents the outline and themes of workshops and parties delivered by GRS.

### Assessment procedures

At baseline and ~7 months later, parents and teachers completed questionnaires and children completed direct assessments of school readiness skills. Teacher questionnaires asked about child social-emotional skills and behavior. Parents completed a set of questionnaires that asked about demographics and language dominance. At post-test, parents responded to questions about their experience with GRS. Research staff handed questionnaires to caregivers at drop off or pick up both at baseline and post-test. Research staff went twice to each classroom to make sure all caregivers received the questionnaires. If children were not present, questionnaires were left in children's cubbies. Parents could fill out the questionnaires in the classroom with the support of research assistants if requested or take them home and bring them back that same week. Parents filled out the surveys in their preferred language. Children were pulled out of their regular classrooms twice on different days by a bilingual, trained research assistant for 20–30 min to participate in assessments of early literacy, oral language, early mathematics skills, and self-regulation.

#### Language dominance

Children who spoke Spanish as reported by their parents were administered three subtests from the *Preschool Language Assessment Scale* (PreLAS 2000; Duncan and De Avila, 1998) to determine language dominance. Children who spoke Chinese as reported by their parents were administered the first 15 items of the *Expressive One Word Picture Vocabulary Test* (EOWPVT; Gardner, 1990) in English and Chinese, and dominance was determined by the higher scoring language. Once language dominance was determined, the dominant language was used for all subsequent assessments. Chinese dominant children only completed the *Head-Toes-Knees-Shoulders* and *toy wrap* tasks in Chinese. Instructions for these self-regulation tasks were translated into Chinese by a native Chinese-speaker who was bilingual in English. Based on language dominance tests, 78.3% children were dominant in English, 17.4% in Spanish, and 4.3% in Chinese.

## Measures

### Parent involvement in GRS

#### Parent attendance at GRS events

Parent attendance was recorded at each of the 11 total GRS events (one orientation, eight workshops through the year, and two parties). Because centers had open enrollment and some families enrolled after GRS started, we calculated a percentage of events attended for each parent based on date of enrollment. This percentage was used in analyses rather than the total number of events attended. In addition, we also calculated attendance separately for the parent orientation, workshops, and parties to examine differences in attendance across different events.

At the end of each workshop, parents completed a survey that asked about the usefulness of different aspects of the workshop (presentation, group discussion, group work, videos; 1 = *very unhelpful* to 5 = *very helpful)*, how prepared they felt to do GRS activities at home (1 = *very unprepared* to 5 = *very prepared)*, and how well they understood the workshop (1 = *very confused* to 5 = *I understand very well)*.

#### Parental time spent doing GRS activities at home

At each GRS party and at the end of the GRS program, parents were asked to fill out a brief survey about time spent doing GRS activities, usage of GRS materials, and satisfaction with materials. We administered this brief survey three times across the year to collect information from as many families as possible. Seventy-seven parents returned this survey at least once. Parents who returned these surveys attended a higher percentage of GRS events (*M* = 33.08; *SD* = 24.79) compared with families who did not return the surveys (*M* = 9.10; *SD* = 12.55), *t*_(131)_ = 7.18, *p* = 0.000. We did not find differences in family or child characteristics between parents who filled out the forms and parents who did not.

As part of this survey, parents were asked how much time they spent doing GRS activities each week (0 = *didn't do GRS activities*, 1 = < *10 min*, 2 = *10 – 30 min*, 3 = *30 – 60 min*, 4 = > *60 min*). Most of the parents who returned the survey did so at the end of the GRS program (*n* = 66, 86%). For parents who returned the survey more than once, we averaged their responses across time points.

In addition, we examined parental perceptions about the activities they did. Parents were asked to rate (1 = *strongly disagree* to 4 = *strongly agree*) the following statements: “The description of the activities is easy to understand” and “The activities in the book are easy to do.”

#### Parental usage of digital materials

To measure usage of digital materials, parents were asked whether they had used the classroom tablet computers or the GRS website to access the videos. Usage of digital materials was dichotomized as 0 = *never accessed GRS videos*, 1 = *accessed GRS videos* (on the tablet and/or the website).

### School readiness skills

#### Early literacy

The *Woodcock-Johnson Test of Academic Achievement (WJ III)/Bateria III Woodcock Munoz* (WJ III; Woodcock et al., 2001) is a widely used, standardized battery of academic skills, with strong validity and reliability in both English and Spanish. The Letter-Word Identification subtest involves identifying printed letters and later reading printed words aloud. The Picture Vocabulary subtest assesses oral language and lexical knowledge. Internal consistency (Cronbach's alpha) for these subtests ranges from 0.81 to 0.98. Test-retest reliability ranges from 0.89 to 0.92 (Bradley-Johnson et al., 2004).

The *Clinical Evaluation of Language Fundamentals—Preschool*−*2* (CELF-P-2 and CELF-P-2-Spanish; Semel et al., 2003) phonological awareness subtests, normed in both English and Spanish, assess rhyming, blending, segmenting, and identifying sounds and syllables in words and sentences. The English version has a test-retest reliability of 0.82–0.86 and a Cronbach's alpha of 0.88. The Spanish version has a test-retest reliability of 0.81–0.93 and internal consistency of 0.82–0.88. Because the Spanish and English forms only have three subscales that match, we created a composite by summing scores on the syllabic blending, sentence segmentation, and syllabic segmentation tests.

#### Early math

The *WJIII/Bateria Woodcock-Munoz* (Woodcock et al., 2001) Applied Problems subtest measures the child's ability to analyze and solve math problems. Initial items involve counting and identifying the number of objects in a picture, eventually progressing to more complex calculations. Cronbach's alpha ranges from 0.90 to 0.92 (Bradley-Johnson et al., 2004).

The *Test of Early Mathematics Ability (TEMA-3*; Ginsburg and Baroody, 2003) measures informal and formal concepts and skills in counting, number-comparison facility, number literacy, number facts, calculation skills, and understanding of number concepts. The TEMA-3 has been normed for children between the ages of 3 and 8 years. The Spanish version was provided to us by the developer of the TEMA-3. Internal consistency reliabilities have been reported all above 0.92 (Ginsburg and Baroody, 2003). Performance on the TEMA-3 is highly correlated with the WJ III Tests of Achievement (Woodcock et al., 2001).

#### Self-regulation

The *Head-Toes-Knees-Shoulders* (HTKS; McClelland et al., 2014) task measures behavioral self-regulation including aspects of executive function (Ponitz et al., 2009; McClelland and Cameron, 2012). This task has been used for children aged 4–8 years. The instructions are available in English and Spanish. Children first follow one of two commands naturally, and then are instructed to respond with a conflicting, non-automatic action. For example, if the administrator says, “Touch your head,” the correct response would be to touch one's toes. On each item, children score 2 points for responding correctly, 1 point for self-correcting, and 0 points for responding incorrectly. In previous research, children who perform better on the HTKS task receive higher teacher and parent ratings of behavioral competence, and fall prekindergarten scores predict achievement level and gains in the spring (McClelland et al., 2007; Ponitz et al., 2009). The HTKS task has demonstrated construct and predictive validity in preschool through first grade, in both English- and Spanish-speaking children (Ponitz et al., 2008; McClelland et al., 2014). It has an inter-rater reliability of 0.98 (Connor et al., 2010).

The *Preschool Self-Regulation Assessment* (PSRA; Smith-Donald et al., 2007) is a brief battery of tasks designed to assess self-regulation in preschool children. It is available in English and Spanish and has been used in both the Chicago School Readiness Project (Smith-Donald et al., 2007) and in the Head Start REDI Program (Bierman et al., 2008a,b). In the toy wrap task, the child is instructed not to peek for 1 min while the researcher noisily wraps a “surprise.” Scores reflect how many times the child peeks and how much time elapses before the child peeks.

#### Social-emotional skills

The *Social Competence and Behavior Evaluation Short Form (SCBE-30;* LaFreniere and Dumas, 1996) is a well-validated, 30-item measure used by teachers to rate children using 6-point Likert scales. It contains the following three scales: Anger/Aggression, Anxiety/Withdrawal, and Social Competence. Scores at preschool-age predict later social competence (Denham et al., 2003). The SCBE-30 has an inter-rater reliability of 0.78–0.91, and Cronbach's alpha ranging from 0.80 to 0.92.

### Dosage of classroom activities

Teachers reported the activities they had completed at least once during a period of 2 weeks in their bi-weekly coaching meeting (1 = *completed activity*, 0 = *didn't complete activity*). The bi-weekly dosage scores were summed across the entire year to create a measure of dosage fidelity that describes the total number of activities teachers have completed at least once. Dosage of classroom activities was used as a covariate in some analyses.

### Analytic approach

To examine the rates of parental attendance, time spent doing activities, and usage of digital content, we computed descriptive statistics for each parent involvement measure. In addition, we examined differences by school and classroom in parent involvement measures. To examine associations between family characteristics and parent involvement, we used zero-order correlations, *t-*tests, and chi-square tests, as appropriate. To examine the associations between indices of parent involvement in GRS (independent variable) and gains in children's school readiness outcomes post-intervention (dependent variable) we conducted multilevel modeling analyses using linear mixed modeling in SAS (version 9.4; SAS Institute, 2014), given that child level data were nested within classrooms.

Separate regression models were conducted to examine associations of parental attendance and time spent on GRS activities with children's school readiness outcomes. Child post-intervention school readiness outcomes were modeled as a function of parent involvement, after adjusting for the baseline value of the same school readiness measure and the time between the baseline and post-intervention assessments. Child age, sex, race, and ethnicity, and indices of family SES (income-to-needs ratio, parental education) were included as additional covariates in these regression models. Covariates that were not significant were dropped from the final models.

Data on parent attendance was available for all parents (*n* = 133). However, data on time spent doing GRS activities and usage of digital materials was only available for 58% of the total sample. In regard to this missing data, the mixed model approach used in this study has the advantage of using full information maximum likelihood (FIML) and thus including all available cases rather than making listwise deletions.

## Results

### Descriptive statistics

#### Parent attendance at GRS events

The majority of parents (69.20%) attended at least one GRS event (see Table 4). Out of the 92 parents that came to GRS events, 31% came to one event, 24% came to two events, 13% came to three events, 10% came to four events, and the remaining 14% came to five events or more. On average, parents attended 21.88% of total offered events (*SD* = 22.92, range 0–90%). There were significant differences in the percentage of events attended by school/center, *F*_(3, 133)_ = 3.20, *p* = 0.03, and by classroom, *F*_(6, 133)_ = 2.77, *p* = 0.01, ranging from 12.14% (*SD* = 8.95) to 33.01% (*SD* = 26.96). Attendance also differed by event. Across all schools, 37.40% parents came to parent orientation, 18.13% to parent workshops, and 31.5% to GRS parties (see Table 3).

**Table 4:** Descriptive statistics for study variables.

	**Time 1**				**Time 2**			
	***n***	***M***	***SD***	**Range**	***n***	***M***	***SD***	**Range**
% GRS events parents attended	–	–	–	–	133	21.88%	22.92%	0–90%
Parental time spent doing GRS activities	–	–	–	–	77	2.00	0.86	0–4
# parents used videos	–	–	–	–	78	43.6%	34	–
WJ Letter-word identification	97	96.73	23.48	0–130	97	100.75	11.36	71–135
WJ Picture vocabulary	98	95.36	13.37	49–114	98	94.25	12.31	49–121
CELF: Phonological awareness	93	3.16	3.48	0–11	93	7.16	3.40	0–12
WJ Applied problems	96	98.68	13.78	61–129	96	102.82	13.26	58–127
TEMA: Math skills	78	89.71	14.70	68–132	78	94.01	15.04	55–127
HTKS	103	5.55	10.53	0–54	103	16.62	17.18	0–54
Toy wrap (number of peeks)	91	1.82	1.97	0–7	91	1.68	2.35	0–10
SCBE-30: Social competence (T)	105	3.22	0.97	1.40–5.70	105	3.82	1.00	2–5.70
SCBE-30: Anxiety (T)	105	1.80	0.69	1–4.90	105	1.97	0.74	1–4.80
SCBE-30: Anger/Aggression (T)	105	1.59	0.67	1–5.70	105	1.78	0.92	1–5.40

*Descriptive statistics for child outcomes only for children who had pretest and posttest data. T, teacher questionnaire; WJ, Woodcock–Johnson Test of Academic Achievement; CELF, Clinical Evaluation of Language Fundamentals, Phonological Awareness Subtest; TEMA, Test of Early Mathematics Ability; HTKS, Head-Toes-Knees-Shoulders; SCBE30, Social Competence and Behavior Evaluation-Short Form*.

Ratings of how helpful a workshop was, how prepared parents felt doing activities, and how clear a workshop topic was consistently averaged 4.4 or higher on a 5 point scale (*SD* = 0.69). Overall, more than 90% of parents who participated in GRS workshops found them helpful and felt prepared to do the activities with their children, as indicated by ratings above of 4 out of 5.

#### Parental time spent doing GRS activities at home

Out of 77 parents who reported on time spent on GRS activities in a week, 6.5% reported they did not have time to do GRS activities, 9.1% reported they spent <10 min, 58.4% spent 10–30 min, 23.4% spent 30–60 min, and 2.6% spent > an hour. On average, parents spent between 10 and 30 min per week doing GRS activities. There were no significant differences in average time spent on activities by classroom, *F*_(6, 133)_ = 0.44, *p* = 0.85, or school, *F*_(3, 133)_ = 0.47, *p* = 0.76. In addition, most parents agreed that activities were easy to understand (76.6%) as well as to implement (80.35%).

#### Parental use of digital program materials

Parental use of videos was slightly higher. Out of 78 parents who answered this question, 43.6% reported having seen videos from the tablet and/or the website. There were no significant differences in use of digital materials by classroom, $\chi_{\left(1\right)}^{2}$ = 3.05, *p* = 0.80, or by school, $\chi_{\left(1\right)}^{2}$ = 2.98, *p* = 0.56.

#### Associations among parent involvement indices

The three parental involvement indices were not significantly inter-correlated. Time spent on GRS activities was not associated with attendance to GRS events, *r* = 0.08, *p* = 0.52. Parents who used the digital materials were not significantly more likely to attend GRS events, *t*_(77)_ = 1.93, *p* = 0.06, or spend more time doing GRS activities at home, *t*_(73)_ = 1.20, *p* = 0.24. These results were likely due to the smaller subset of parents who reported on time spent doing activities at home and use of digital materials being higher in parental attendance compared to parents who did not report on these variables. Thus, there was likely restricted variability in parental time spent doing activities and use of digital materials.

### Family characteristics and parent involvement in GRS

#### Parent attendance at GRS events

Out of a broad range of family characteristics, including income-to-needs ratio, maternal education, immigration history, race, language, and use of social services, only parental employment and food stamps receipt were significantly associated with parent attendance at GRS events. Working parents attended 41% of GRS events (*SD* = 26.56) whereas non-working parents attended 25% of GRS events (*SD* = 23.19), *t*_(66)_ = 2.59, *p* = 0.02. Families receiving food stamps (Supplemental Nutrition Assistance Program or SNAP) attended 25% (*SD* = 23.09) of events while families not receiving food stamps attended 40% of events (*SD* = 26.17), *t*_(66)_ = 2.53, *p* = 0.02.

#### Parental time spent doing GRS activities at home

Only family income-to-needs ratio was positively and significantly associated with parental time spent doing GRS activities per week, *r* = 0.25, *p* = 0.04.

#### Parental use of digital program materials

Having the father present in the child's life was significantly associated with use of digital content, with 54% of families with the father present and 13% with the father not present in the child's life having used the digital materials, $\chi_{\left(1\right)}^{2}$ = 4.74, *p* = 0.03.

### Parent involvement in GRS and gains in children's school readiness skills

We examined the associations between child/family characteristics and children's post-intervention math, literacy and self-regulation skills to determine which covariates should be included in subsequent regression models. Four of these characteristics (child ethnicity, sex, dominant language, and respondent education) were identified as predictors of some child outcomes. Children whose primary caregiver had at least a high school diploma scored higher on picture vocabulary [*t*_(73)_ = 2.10, *p* = 0.01]. Hispanic/Latino children scored lower on letter word identification [*t*_(73)_ = 2.66, *p* = 0.002], applied problems [*t*_(73)_ = 3.05, *p* = 0.001], and math skills [*t*_(69)_ = 3.32, *p* = 0.001]. Children whose dominant language was Spanish scored lower on letter word identification [*t*_(109)_ = 2.77, *p* = 0.03] picture vocabulary [*t*_(109)_ = 4.37, *p* = 0.00], phonological awareness [*t*_(107)_ = 2.87, *p* = 0.01], applied problems [*t*_(109)_ = 4.96, *p* = 0.00], math skills [*t*_(99)_ = 4.32, *p* = 0.003], HTKS [*t*_(111)_ = 2.42, *p* = 0.02], and showed lower levels of anger [*t*_(100)_ = −2.61, *p* = 0.01]. Girls scored higher on applied problems [*t*_(109)_ = 2.16, *p* = 0.03], social competence [*t*_(115)_ = 3.50, *p* = 0.001], and scored lower on problem behavior [*t*_(115)_ = −2.41, *p* = 0.02], and peeked less on the toy wrap task [*t*_(90)_ = −2.42, *p* = 0.04]. Covariates that were found to be significant were included in the subsequent regression models.

#### Regression models

Results are presented in Tables 5, 6. Higher parental attendance at GRS events was significantly associated with higher early literacy (picture vocabulary, letter-word identification, phonological awareness), self-regulatory skills (toy wrap task), and social competence even after controlling for the nesting of children within classrooms, baseline skill levels, and other relevant covariates (see Table 5). We also examined the association between parental attendance and child school readiness outcomes after including GRS dosage in the classroom as a covariate. Most associations remained the same (picture vocabulary, β = 0.11, *p* = 0.04; phonological awareness, β = 0.17, *p* = 0.03; toy wrap task, β = −0.54, *p* = 0.01; and social competence, β = 0.14, *p* = 0.06). The association between parental attendance and letter-word identification became non-significant (β = 13, *p* = 0.14)

**Table 5:** Parent attendance effects on children's post-intervention school readiness.

**(A) Parent attendance effects on post-intervention language, pre-literacy, and math outcomes**.															
	**Letter-word identification**			**Picture vocabulary**			**Phonological awareness**			**Applied problems**			**Math skills**		
	β	***SE***	***p***	β	***SE***	***p***	β	***SE***	***p***	β	***SE***	***p***	β	***SE***	***p***
Baseline	0.42	0.08	<0.001	0.83	0.07	<0.001	0.41	0.11	0.00	0.66	0.08	<0.001	0.70	0.09	<0.001
Child race	−0.25	0.09	0.01	–	–	–	–	–	–	−0.22	0.08	0.01	0.07	0.10	0.46
Child sex	–	–	–	–	–	–	–	–	–	0.03	0.07	0.61	–	–	–
Child language	0.22	0.14	0.11	−0.55	0.10	<0.001	0.05	0.16	0.78	−0.02	0.13	0.89	−0.18	0.11	0.12
Parental education	–	–	–	0.01	0.08	0.93	–	–	–	–	–	–	–	–	–
Attendance	0.16	0.07	0.03	0.13	0.06	0.03	0.19	0.08	0.03	0.00	0.07	1.00	0.04	0.07	0.56
**(B) Parent attendance effects on post-intervention self-regulation and social-emotional outcomes**.															
	**HTKS**			**Toy wrap (#peeks)**			**Social competence (T)**			**Anger (T)**			**Anxiety (T)**		
	β	***SE***	***p***	β	***SE***	***p***	β	***SE***	***p***	β	***SE***	***p***	β	***SE***	***p***
Baseline	0.48	0.11	<0.001	0.45	0.11	0.00	0.54	0.10	<0.001	0.90	0.10	<0.001	0.64	0.09	<0.001
Child race	–	–	–	–	–	–	–	–	–	–	–	–	–	–	–
Child sex	–	–	–	−0.19	0.10	0.08	0.19	0.08	0.02	–	–	–	–	–	–
Child language	−0.07	0.12	0.56	–	–	–	−0.04	0.11	0.68	–	–	–	0.02	0.10	0.88
Parental education	–	–	–	–	–	–	–	–	–	–	–	–	–	–	–
Attendance	−0.05	0.09	0.58	−0.21	0.09	0.02	0.18	0.08	0.04	−0.09	0.08	0.24	−0.05	0.08	0.49

*Models control for time between pre-test and post-test and child age with no difference in results. Therefore, child age and time between pre and posttest estimates are not included in the table. We did not control for time between assessments for SCBE subscales because there was no variation. To preserve power, we only include child and family demographic variables that showed a significant correlation with child outcome in bivariate analysis. (T) teacher-rated*.

**Table 6:** Parental time spent on GRS activities effects on children's post-intervention school readiness.

**(A) Parental time spent on GRS activities effects on post-intervention language, pre-literacy, and math outcomes**															
	**Letter-word identification**			**Picture vocabulary**			**Phonological awareness**			**Applied problems**			**Math skills**		
	***β6***	***SE***	***p***	**β**	***SE***	***p***	**β**	***SE***	***p***	**β**	***SE***	***p***	**β**	***SE***	***p***
Baseline	0.51	0.08	<0.001	0.78	0.10	<0.001	0.34	0.12	0.01	0.71	0.10	<0.001	0.80	0.11	<0.001
Child race	−0.24	0.09	0.01	–	–	–	–	–	–	−0.15	0.09	0.13	0.11	0.12	0.40
Child sex	–	–	–	–	–	–	–	–	–	0.21	0.08	0.01	–	–	–
Child language	0.15	0.13	0.25	−0.53	0.13	<0.001	0.21	0.18	0.25	−0.11	0.14	0.45	−0.11	0.14	0.42
Parental education	–	–	–	0.02	0.09	0.80	–	–	–	–	–	–	–	–	–
Times spent on GRS activities	0.19	0.08	0.03	−0.01	0.10	0.93	−0.02	0.11	0.89	0.21	0.08	0.01	0.10	0.09	0.29
**(B) Parental time spent on GRS activities effects on post-intervention self-regulation and social-emotional outcomes**															
	**HTKS**			**Toy wrap (#peeks)**			**Social competence (T)**			**Anger (T)**			**Anxiety (T)**		
	**β**	***SE***	***p***	**β**	***SE***	***p***	**β**	***SE***	***p***	**β**	***SE***	***p***	**β**	***SE***	***p***
Baseline	0.49	0.12	0.00	0.31	0.12	0.02	0.59	0.11	<0.001	0.72	0.15	<0.001	0.55	0.08	<0.001
Child race	–	–	–	–	–	–	–	–	–	–	–	–	–	–	–
Child sex	–	–	–	−0.21	0.14	0.13	0.26	0.10	0.01	–	–	–	–	–	–
Child language	−0.02	0.13	0.88	–	–	–	−0.05	0.12	0.67	–	–	–	0.10	0.11	0.37
Parental education	–	–	–	–	–	–	–	–	–	–	–	–	–	–	–
Times spent on GRS activities	−0.14	0.10	0.15	0.02	0.14	0.90	−0.13	0.09	0.15	0.00	0.11	0.98	0.02	0.08	0.83

*Models control for time between pre-test and post-test and child age with no difference in results. Therefore, child age and time between pre and post-test estimates are not included in the table. We did not control for time between assessments for SCBE subscales because there was no variation. To preserve power we only include child and family demographic variables that showed a significant correlation with child outcome in bivariate analysis. (T) teacher-rated*.

The more time parents spent on GRS activities in a given week, the more gains were observed in letter-word identification and applied problems controlling for baseline skill levels, relevant covariates, and nesting effects (see Table 6). Associations between time spent on GRS activities and child school readiness outcomes did not change when GRS classroom dosage was added as a covariate (letter-word identification, β = 0.24, *p* = 0.03) and applied problems, β = 0.26, *p* = 0.01). Effect sizes for these results were small for parental attendance (η_*p*_^2^: 0.02–0.08) and medium for parental time spent doing activities (η_*p*_^2^: 0.10–0.15). Parental use of digital program materials was not associated with gains in any child outcome.

## Discussion

The goals of this study were to examine how parents engage in the GRS intervention, associations between family characteristics and parent involvement in GRS, and associations between parent involvement and gains in children's school readiness skills over the preschool year. On the whole, few family characteristics were significantly associated with parental involvement in GRS. However, there was a tendency for markers of relatively higher SES to be linked with greater parental involvement in GRS. Findings also indicated that greater parent involvement in GRS may lead to more positive gains in children's school readiness skills.

Two-thirds of parents attended at least one GRS event at the school, suggesting that family-oriented school readiness interventions like GRS may be feasible for low-income families. Nonetheless, parental attendance to events was inconsistent, with few parents repeatedly attending GRS events. Other prevention programs that offer services for Head Start families have found similar results (Gross et al., 2009). For example, Head Start parents were found to attend less than two workshops out of nine offered as part of *The Companion Curriculum* intervention (Mendez, 2010). Interestingly, we found differences in parental attendance based on the type of event. While parent workshops consistently had low attendance (average attendance to workshops was 18%), attendance at GRS parties in the classroom was higher (30%). We conducted 10 focus groups with 40 parents at the end of the intervention to understand the barriers to, and the facilitators of, program participation. Results indicated that for some parents the GRS activities were self-explanatory and given their limited time GRS program materials were enough for them to understand how to engage in learning activities with their children. This may explain the lack of consistent attendance to workshops. In addition, differences in when events were scheduled may explain these differences in parental attendance to GRS events, with workshops taking place in the morning and parties in the afternoon. It may also be that an event described as a “party” is inherently more appealing than a “workshop.” Focus group participants also pointed out that they liked the opportunity to engage and play with their children during the parties. Regardless, offering a variety of events at different times and in different formats may be an effective means of increasing parent involvement in early interventions.

Close to 26% of parents reported using GRS activities 30 min per week or more, and almost 60% of parents reported using GRS activities between 10 and 30 min per week. Thus, spending around 30 min per week engaging in simple and fun activities may be feasible for most families. However, results may be biased toward more engaged parents given that data were available for less than two thirds of the sample, and this subsample attended more GRS events. In addition, GRS activities are intended to be used repeatedly and integrated into daily routines. Thus, future research should explore more nuanced measures including whether parents incorporate program activities into their daily routines.

Almost 50% of parents reported having used the videos that were provided for each parent-child activity to support the implementation of the activity at home. However, this percentage may be lower if data were available for the whole sample. Nonetheless, these results suggest that offering digital program materials may be a practical, cost-effective way to engage parents. However, more research is needed to understand parents' preferences in regard to using internet-based materials and whether those materials by themselves would be enough to produce intervention effects.

Findings from this study indicated a tendency for family characteristics associated with lower SES to be associated with reduced parental involvement in the GRS intervention. More specifically, unemployed parents attended fewer GRS events than employed parents. In addition, families receiving SNAP (food stamps) attended fewer GRS events compared to those not receiving SNAP. Similarly, lower family income-to-needs ratio was significantly associated with less time spent doing GRS activities at home. These findings suggest the importance of identifying families with the greatest economic need who may benefit from extra support to overcome any barriers to parental participation in early interventions (Cooper et al., 2010; Dawson-McClure et al., 2015). Previous research has suggested that parents with lower educational attainment tend to provide fewer opportunities for learning at home and spend less time engaging in learning activities (Fantuzzo et al., 2000; Kalil et al., 2012; see also Hayes et al., 2016). In the present study, parental education was not significantly associated with parental involvement in GRS, consistent with the notion that the simple and easy design of GRS activities may allow parents with a range of educational backgrounds to implement them at home. In addition, contrary to previous research (Hindman et al., 2012), we did not find differences in parental involvement by ethnicity. The use of parents' preferred language and having staff with similar ethnic background might have helped accommodate parents with different cultural backgrounds (Murry et al., 2004).

Another important finding that emerged was that greater parental involvement in GRS was significantly linked with more positive gains in children's early literacy, math, and self-regulatory skills across the preschool year, even after controlling for family characteristics. Children whose parents attended more GRS events showed more growth in pre-literacy, language, and delay of gratification skills across the preschool year. Similarly, greater parental time spent doing GRS activities at home was associated with greater gains in children's early literacy and math skills. However, it is important to note that these findings do not stem from a randomized experiment, but rather reveal differences among parents receiving the intervention.

These results highlight the importance of offering parents different opportunities to attend in-person events where they can learn strategies to support their children's school readiness skills. Attending GRS events may improve parents' knowledge of how to support school readiness as well as enhance parent-child interactions. Time spent at home doing GRS activities also appears to contribute to child outcomes, especially to gains in early math skills. It might be that parents reporting more time doing GRS activities are indeed spending more time doing math activities. Future research should explore the use of different types of activities at home and their impact on school readiness. These findings suggest that interventions aimed at improving school readiness should incorporate activities and materials that parents can use at home with their children. These strategies may be more cost-effective and scalable, and may offer parents who cannot participate in school events the opportunity to engage in the intervention and promote their children's development and education.

Interestingly, despite parental attendance being associated with gains in children's language/literacy and delay of gratification skills, parental attendance was not significantly associated with children's early math skills. Attendance at math workshops was the lowest and could explain the lack of association with math gains. Previous research with low-income families has suggested that parents of preschool children are more driven to engage in literacy activities than math activities (Cannon and Ginsburg, 2008). This may be related to parents feeling uncertain about mathematics expectations for preschoolers, lacking confidence in their ability to teach math to their young children, or beliefs that the school has the primary responsibility of teaching math (Cannon and Ginsburg, 2008; Maloney et al., 2015).

Future research should explore strategies (e.g., text reminders) to support and increase the time parents spend at home engaged in learning activities with their children (Gennetian et al., 2017) and decrease barriers to attending program events that promote parent involvement. Future parent involvement research with a larger sample should explore the unique contributions of attending school-based workshops and using home-based learning activities.

## Limitations

The findings of this study must be considered in light of several limitations. First, measures of parental satisfaction with the GRS intervention and time spent doing GRS activities at home were based on parents who responded to surveys. Therefore, responses may have been affected by social desirability. In addition, given that 58% of families returned these surveys and that parents who responded to surveys had higher attendance to events than non-respondents, these results may be biased toward more engaged families. More objective measures might have resulted in different associations. Methods such as time-diaries should be considered in future studies as they might provide data that is more precise. This makes it important to take into account results for parent attendance, as this measure of parent involvement was not biased in this way; findings for this measure were similar to those for the other measures of parent involvement. Second, the smaller sample size for these survey-based measures may have reduced power to detect effects. Therefore, these associations should be viewed as exploratory in nature and cannot be generalized to other samples. Third, despite the longitudinal design of this study, we cannot make inferences about the direction of causality from these data. We did not account for general measures of home-based and school-based parental involvement and quality of parent-child interactions that may explain variability in parent involvement with GRS and child outcomes. Parents with higher levels of involvement at the beginning of the preschool year could be more inclined to engage with a school readiness intervention like GRS. In addition, rates of children's preschool attendance could partially explain differences in parental attendance to GRS events. Fourth, although we controlled for the potential effects of children being nested within classrooms and for dosage of GRS in the classroom, we did not specifically examine the role of other variables related to GRS implementation in the classroom such as child engagement with GRS classroom activities.

## Conclusion

In this study, higher levels of parent involvement with the GRS intervention were found to be associated with greater gains in early literacy, math, and self-regulatory skills among preschoolers from socioeconomically disadvantaged families. Therefore, enhancing parent involvement in early school readiness interventions may lead to improved developmental gains for socioeconomically disadvantaged children. These results underscore the importance of offering parents various opportunities for involvement in school readiness interventions. GRS offers parents easy to understand materials and flexible options to promote child development using playful activities. By assessing and enhancing various aspects of parent involvement in early interventions, we may discover new ways of improving school readiness that are well-matched to the diverse population of families who participate in these interventions.

## Author contributions

MM conceptualized the study, carried out the analyses, drafted the initial manuscript, and approved the final manuscript as submitted. EM reviewed the analyses, reviewed and revised the manuscript, and approved the final manuscript as submitted. KR edited the manuscript. CL, KN, and HD reviewed and revised the manuscript, and approved the final manuscript as submitted.

### Conflict of interest statement

The authors declare that the research was conducted in the absence of any commercial or financial relationships that could be construed as a potential conflict of interest.
